# Conceptual and practical classification of research reviews and other evidence synthesis products

**DOI:** 10.4073/cmdp.2018.1

**Published:** 2018-07-31

**Authors:** Julia H. Littell

## ABSTRACT

This paper builds on existing taxonomies and typologies of research reviews to create an inclusive conceptual framework for classifying diverse evidence synthesis methods. Previous typologies are incomplete and there is little consistency among them in descriptions of review and synthesis methods. A more inclusive framework may promote better understanding, wider applications, and more judicious use of synthesis methods.

## INTRODUCTION

Growing interest in evidence synthesis has stimulated development of novel synthesis methods. Along with valuable new tools and approaches for handling different kinds of questions and data, the proliferation of synthesis methods has produced some confusion. Some review terms are applied in idiosyncratic or indiscriminate ways, some methods are ill‐defined, and the fit between questions and synthesis methods is often under‐emphasized or unclear. In this paper, I examine the terms and typologies used to describe diverse synthesis methods, identify distinct and overlapping types of synthesis, and consider the goodness of fit between different synthesis questions and methods. This paper is motivated by interest in supporting judicious selection of synthesis methods that are fit for different purposes.

## BACKGROUND

The production of systematic reviews and meta‐analyses in the social and health sciences began to increase exponentially in the early 1990s, but this trend is overshadowed by even steeper rises in the production of non‐systematic reviews (Bastian, Glasziou, & Chalmers, 2010; Littell, 2016). This rise in research synthesis activity is due, in part, to increased research output (Bastian et al., 2010) and awareness that synthesized evidence is more robust and potentially more useful than results of individual studies (Grimshaw, Eccles, Lavis, Hill, & Squires, 2012).

Beyond meta‐analyses of data from randomized controlled trials on effects of interventions, systematic review methods have been extended to diverse types of research questions and data. New search, appraisal, and synthesis methods have emerged within and across disciplines. On one hand, such diverse methods are needed to synthesize different types of evidence on the wide range of topics that are important to funders, policy makers, consumers, and researchers. However, stakeholders are now faced with a rather bewildering array of review and synthesis products, including: systematic reviews, meta‐analyses, network meta‐analyses, rapid reviews, realist reviews, qualitative evidence syntheses, scoping reviews, and evidence and gap maps.

## CONFUSING TERMINOLOGY

For purposes of this paper, I use the terms “research review” and “evidence synthesis” interchangeably to refer to a wide range of approaches to the identification, appraisal, and analysis of empirical research; these efforts may or may not include the synthesis of results across studies.

There is little consensus on the meanings of terms commonly used to describe research reviews. This is similar to the terminological confusion that appears in the literature on designs for primary research (e.g., the terms “controlled study” and “quasi‐experimental design” have multiple meanings). In the research literature, this confusion is known as the jingle/jangle fallacy (Pedhazur & Schmelkin, 1991).

### The jingle fallacy

The jingle fallacy is the erroneous belief that two different things are the same because they are given the same name. As a prime example, the term “systematic review” has taken on multiple meanings in recent years. According to the Cochrane Handbook,

A systematic review attempts to collate all empirical evidence that fits pre‐specified eligibility criteria in order to answer a specific research question. It uses explicit, systematic methods that are selected with a view to minimizing bias, thus providing more reliable findings from which conclusions can be drawn and decisions made (Higgins & Green, 2011).

With these twin emphases on comprehensive coverage and accuracy, systematic reviews are viewed as the most reliable sources of evidence for practice and policy (Clarke, 2011). However, the term “systematic review” has been applied to a wide range of review methods, including approaches that are neither comprehensive nor reliable. Some reviews are described as systematic solely because they used keyword searches of an electronic database. Other so‐called “systematic reviews” follow explicit rules and procedures that have been shown to invite bias and error (e.g., systematically excluding eligible unpublished studies, or relying on a single coder).

Similarly, the term “rapid review” has been used to describe diverse approaches (Polisena et al., 2015), and “scoping reviews” use a wide range of methods (Pham et al., 2014). When the same terms are applied to disparate approaches, readers may not be aware of important distinctions between them.

### The jangle fallacy

The jangle fallacy is the erroneous belief that two identical (or nearly identical) things are different because they are given different names. For example, the terms used to describe reviews of existing reviews include: overviews, umbrella reviews, systematic reviews of reviews, and meta‐reviews – although distinctions between these terms are neither consistent nor clear (Ballard & Montgomery, 2017).

To address this confusion, it is necessary to look beyond the current lexicon to consider important elements of diverse approaches to the identification, analysis, and synthesis of empirical evidence. To do this, we shall examine and build on existing classification systems.

## CLASSIFICATION SYSTEMS

Typologies and taxonomies are two different types of classification systems (Bailey, 1994). A taxonomy uses empirical observations to classify items into categories, while a typology is based on theoretical or conceptual distinctions. Either approach can employ multi‐dimensional structures. Classification systems are useful for organizing information and identifying important dimensions on which members of the class (in our case, research reviews) may vary (Collier, LaPorte, & Seawright, 2012). However, attempts at classification risk the imposition of artificial structures on phenomena that may not fit neatly into prescribed categories; and multi‐dimensional classification schemes do not necessarily solve this problem.

Like designs for primary research (see Beissel‐Durrant, 2004; Luff, Byatt, & Martin, 2015) and public policies (see Smith, 2002), research reviews defy a basic rule of classification, which stipulates that categories must be both exhaustive (covering all instances) and mutually exclusive (with no member belonging to more than one category). In other words, in classical set theory there must be one category – but only one category – for each member (Bailey, 1994). More flexible approaches to classification are possible, when we use “fuzzy sets” that don't have clear or arbitrary boundaries and allow each member of a class to take on multiple values or positions within a category.

Cooper (1988) and Gough, Thomas, and Oliver (2012) noted that research reviews differ and overlap in so many aspects that a simple taxonomy or typology of reviews is not possible. They identified key characteristics or dimensions on which reviews vary.

### Key characteristics of reviews

**Figure 1:**
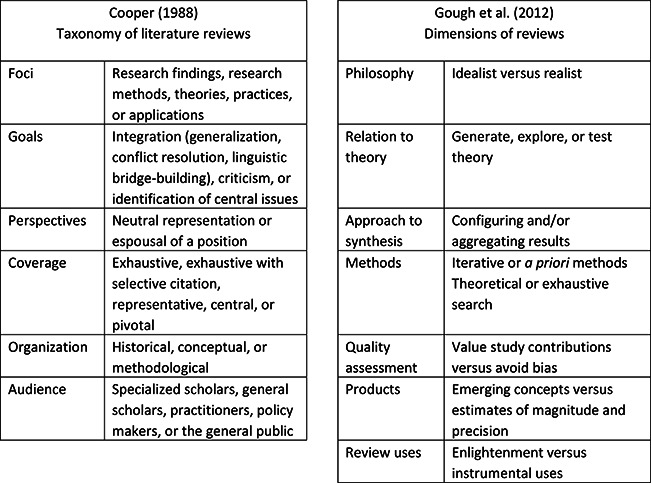
Comparison of Cooper's (1988) taxonomy and Gough et al. (2012) dimensions of reviews.

Harris Cooper was one of the first social scientists to suggest that research reviews should follow basic steps in the scientific process. Cooper (1988) developed and assessed a taxonomy of reviews based on existing literature, unstructured interviews with 14 scholars in psychology and education, reliability checks on independent classifications of 37 reviews, and a survey completed by 108 review authors. As shown in Figure 1, Cooper (1988) characterized literature reviews according to their foci, goals, perspectives, coverage, organization, and audience. Using this taxonomy, Cooper found low initial agreement on independent classifications of reviews, in part because many reviews have multiple foci or multiple goals. However, when reviewers were asked to describe their own reviews using Cooper's taxonomy, few objected to these categories or suggested new ones.

Gough, Thomas, and Oliver (2012) provided another classification system, focusing on variations in the aims and approaches, structures and components, and breadth and depth of research reviews. They identified several dimensions (or continua) on which reviews vary, including philosophy, relation to theory, approaches to synthesis, use of iterative or a priori methods, search strategies, approaches to quality assessment, products, and uses.

As shown in Figure 1, there is little overlap between Cooper's (1988) taxonomy and the dimensions identified by Gough and colleagues (2012).

### Typologies of reviews

Several typologies of research reviews and emerging knowledge synthesis methods are shown in Table 1. (1) Grant and Booth (2009) identified 14 types of reviews and described the methods commonly used within each approach for searching, appraisal, synthesis, and analysis of evidence. (2) The Royal Pharmaceutical Company (2011) described key features, similarities, and differences of eight types of reviews. (3) Tricco and colleagues (2016) conducted a scoping review of “emerging knowledge synthesis methods” in health, education, sociology, and philosophy. They identified 25 distinct synthesis methods; of these, 12 provided guidance for the entire process (including search, critical appraisal, and synthesis; these are shown in Table 1) and 13 provided guidance for synthesis only. (4) Perrier and colleagues (2016) found more than 600 articles on “emerging knowledge synthesis methods” published in 330 different journals, with steady increases in the number of these publications after 2003; the most commonly used approaches are shown in Table 1.

**Table 1:** Typologies of research reviews and emerging knowledge synthesis methods

Label	Grant & Booth (2009)	Royal Pharmaceutical Society (2011)	Tricco et al. (2016)	Perrier et al. (2016)	Other labels
Content analysis				X	
Concept synthesis			X		
Critical interpretive synthesis			X	X	
Critical review	X				
Integrative review			X	X	
Literature review	X			X	
Mapping review or systematic map	X				Evidence and gap map (3ie, Campbell)
Meta‐analysis	X	X			
Meta‐ethnography			X	X	
Meta‐interpretation			X		
Meta‐narrative			X	X	
Meta‐study			X	X	
Meta‐summary or meta‐aggregation			X		
Meta‐synthesis			X	X	
Mixed studies or mixed methods review	X	Combine quantitative & qualitative studies	X	X	Multi‐level vs. parallel syntheses (Noyes et al., 2011)
Narrative synthesis		X	X		
Overview	X				
Qualitative review or evidence synthesis	X	X			
Rapid review	X	Rapid evidence review			Rapid evidence assessment (CEBMa)
Realist review		X	X	X	
Scoping review	X	X			
State‐of‐the‐art review	X				
Systematic review (SR)	X	X		SR w/ novel methods	10 SR types (Munn et al., 2018)^a^
Systematic search and review	X				
Systematized review	X				
Thematic analysis				X	
Umbrella review	X				

a Munn et al. (2018) classified SRs by foci: effectiveness, experiential (qualitative), costs/economic evaluations, prevalence and/or incidence, diagnostic test accuracy, etiology and/or risk, expert opinion or policy, psychometric, prognostic, and methodology

In contrast to typologies that focus on review methods, Munn and colleagues (2018) propose a typology of systematic reviews (in the medical and health sciences) that categorizes review topics. Their typology includes 10 foci of systematic reviews: effectiveness, experiential (qualitative), costs/economic evaluations, prevalence and/or incidence, diagnostic test accuracy, etiology and/or risk, expert opinion or policy, psychometric, prognostic, and methodology.

Diverse methodologies for the synthesis of qualitative evidence have also been identified by several teams. As shown in Table 2, four teams of authors have identified a total of 23 distinct qualitative evidence synthesis (QES) methodologies. Some of these methodologies (e.g., meta‐ethnography and qualitative comparative analysis) include multiple methods for analysis or synthesis and some analysis/synthesis methods (e.g., thematic analysis) are shared by different methodologies.

**Table 2:** Methodologies for synthesizing qualitative evidence

Label	CRD (2009)	Barnett‐Page & Thomas (2009)	Saini & Shlonsky (2012)	Booth et al. (2016)
Case survey	X			
Concept analysis				X
Content analysis	X	X		
Critical interpretive synthesis		X		X
Ecological triangulation		X		X
EPPI‐Centre methods				X
Framework synthesis		X		
Grounded theory, constant comparative method	X	X	X	X
Meta‐aggregation				X
Meta‐ethnography^a^	X	X	X	X
Meta‐interpretation		X	X	X
Meta‐narrative		X		X
Meta‐study		X	X	X
Meta‐summary			X	X
Meta‐synthesis			X	
Miles & Huberman's analysis				X
Narrative summary				X
Narrative synthesis	X	X		X
Qualitative comparative analysis^b^	X			
Qualitative interpretive meta‐synthesis				X
Qualitative meta‐summary		X		
Realist synthesis				X
Thematic synthesis	X	X		X

a Meta‐ethnography includes several synthesis methods including: reciprocal translational analysis, refutational synthesis, line of argument analysis (Barnett‐Page & Thomas, 2009) and thematic analysis (Booth et al., 2016).

b There are three types of qualitative comparative analysis (QCA): crisp‐set, multi‐valued, and fuzzy set.

Authors of previous review typologies note the overlap between review types, lack of explicit methodologies for some approaches (Grant & Booth, 2009), and an overall “lack of guidance on how to select a knowledge synthesis method” (Tricco et al., 2016, p. 19).

Tables 1 and 2 suggest that each typology is incomplete. There is little overlap in the review types – or terminology used to describe reviews – across these papers.

## CONCEPTUAL CLASSIFICATION OF RESEARCH REVIEWS

I aim to develop a more comprehensive system for classifying research reviews across topics, methods, and disciplines. This classification schema is informed by the taxonomies and typologies described above, including pivotal examples (Cooper, 1988; Grant & Booth, 2009; Gough et al., 2012), empirical studies (Tricco et al., 2016; Perrier et al. 2016), and divergent approaches (e.g., Munn et al., 2018).

Instead of a typology of “crisp sets” (mutually exclusive, exhaustive categories), this schema uses multiple criteria (dimensions) and recognizes that reviews can take on more than one value or position within each set of criteria. For example, reviews can have multiple goals. This multi‐dimensional, conceptual schema reflects the inherent complexity and flexibility of research reviews. A summary of the schema is shown in Appendix A and discussed below.

Embedded within the following discussion of these dimensions and characteristics are several emboldened terms; these are terms that are often used to describe certain “types” of reviews; it will become clear that these commonly‐used labels are incomplete and rather idiosyncratic descriptors.

1. *Domain.* Review topics fall within the following broad domains (see Table 3).
  1.1 *Conditions* of interest include social, cultural, economic, environmental, political, medical, cognitive, behavioral, emotional, and/or relational phenomena such as attributes, perceptions, experiences, situations, problems, and disorders.
  1.2 *Interventions* are practices, programs, and policies that attempt to change one or more conditions. Reviews can focus on implementation, acceptability, utilization, complexity, equity, theories of change, outcomes, impacts, costs, replication, and/or dissemination of interventions. Barriers and facilitators to implementation, uptake, and effectiveness may be of interest, along with adherence to and adaptability of interventions in different contexts.
  1.3 *Methodology* reviews are a form of meta‐research, which can consider qualities of research methods, measures, and/or reports.
2. *Topics*. Review topics can be classified according to the kinds of inferences that are sought. Inferences are conclusions based on logic and evidence. As shown in Table 4 and discussed below, different kinds of inferences have different logical and evidentiary requirements. To some extent, these requirements are cumulative, in that later topics tend to build on the requirements of earlier ones. Well‐conceived reviews always address clear questions within a topic (Mulrow, 1987). Examples of questions for evidence synthesis within various topics and domains are shown in Table 3.
  2.1 *Constructs and categories.* To synthesize data about constructs and categories, we need reliable and valid measures or thick descriptions. Relevant topics in each of our three domains include diagnostic categories, treatment typologies, and properties of measurement instruments. Reviews can synthesize data on how people view, understand, or experience a condition or intervention. We might ask how a construct is defined or measured (what nominal and operational definitions are used?) What are the characteristics or properties of a construct (categories, level of measurement, underlying dimensions)? Methodological reviews can assess various properties of measurement instruments including reliability, validity, sensitivity, specificity, and underlying dimensions.
  2.2 *Rates and trends* include studies of incidence and prevalence, other proportions, ratios, averages, and variations. With clear constructs in mind and reliable and valid measures, we can synthesize descriptive data on the incidence or prevalence of a problem or condition (e.g., a disease, crime, school failure, child abuse, poverty). For continuous variables of interest (e.g., household income, years of school completed), we can analyze and synthesize data on averages and distributions. We can also examine changes in rates, averages, or distributions over time, in different places or contexts, or across subgroups.
  2.3 *Associations* between constructs of interest are typically assessed with bivariate and multivariate analyses, which can be synthesized in meta‐analysis. Primary studies and meta‐analyses will need sufficient statistical power to detect associations between variables if these associations exist. Reviews of research on binary gender differences are examples of associational reviews.
  2.4 *Predictions*. In addition to establishing associations between variables, predictions require information on time order, usually in the form of longitudinal data. Reviews in this category can identify risk and protective factors, examine the predictive validity of diagnostic or prognostic tests, or identify outcomes (but not effects or impacts) of different interventions.
  2.5 *Causes and effects*. Reviews of research on the etiology and outcomes of various conditions, belong in this category, along with reviews on the impacts of interventions. In addition to establishing correlation and time order (prediction), reviews in this category will include studies that attempt to rule out rival plausible explanations for causal relationships. In intervention research, this is often done with randomized controlled trials or high‐quality quasi‐experimental designs (Shadish, Cook, & Campbell, 2002). Relevant questions concern the efficacy, effectiveness, and comparative effectiveness of interventions, along with questions about mediators and moderators of these effects. Logic models or theories of change are useful in these reviews. Intervention research on costs effectiveness and cost‐benefit analysis also belongs in this category, as it relies on causal inferences. Methodological reviews can include studies of elements of research design (e.g., as moderators of effect size), features of studies that are associated with reductions in the risk of bias or error, along with methods for analysis and synthesis of data involving complex networks or causal models.
  2.6 *Applications.* Reviews can explore the generalizability of empirical constructs and patterns across studies, settings, populations, cultures, and geopolitical contexts; replication and transportability of interventions; and external validity of research measures and methods. Inferences about generalizability rely on probability sampling methods and inferential statistics or on the proximal similarity of study samples and target populations (Shadish, 1995).
3. *Goals* reflect the aims of a review and the tasks that reviewers expect to accomplish in relation to the topics and questions they have chosen. Well‐conceived reviews have explicit objectives within one or more of the following categories.
  3.1 *Configure information*. Reviews can identify patterns and main themes without necessarily synthesizing study results. For example, **scoping reviews** identify existing studies and **evidence and gap maps** configure information on available studies in matrices (e.g., indicating the types of interventions and outcomes that have been studied); these approaches can inform decisions about future primary studies and syntheses.
  3.2 *Aggregate or synthesize* results. Cooper (1998) suggested that synthesis involves building general statements from specific instances, resolving apparent contradictions by proposing new explanations, and linguistic bridge‐building to unite ideas or observations that may have been expressed differently in different studies. Meta‐analytic tools for these activities include pooling effect sizes across studies, identifying moderators that may account for heterogeneous results, and converting original data to standard metrics. Reviews often combine aggregation and configuration activities in attempts to explore or resolve apparent contradictions across studies.
  3.3 *Appraise study qualities*. Some reviews assess or criticize studies using explicit or implicit external criteria (Cooper, 1988).
  3.4 *Maintain a neutral stance or espouse a position* (Cooper, 1988). Reviews that eschew *a prior* positions on key issues can avoid conflicts of interest and use **systematic review** methods to minimize bias and error at each step in the review process.
  3.5 *Generate, explore, or test theory or hypotheses.* Reviews can consider theories of etiology, theories of change, and alternative plausible explanations.
  3.6 *Inform practice, policy, and/or further research.*
  Note that any one or more of these goals could be applied to any topic or question in Table 3. For example, reviewers can configure information (perhaps in evidence and gap maps) on existing studies of: the incidence or prevalence of a condition, treatment participation, associations, comparative effectiveness of interventions, or the predictive validity of prognostic tools; the same topics can be addressed in reviews that synthesize results across studies.
4. *Planning.*
  4.1 *A priori* plans are often described in a protocol, with the expectation that any changes in or deviations from these initial plans will be explained in the final report. This approach is related to the goal of minimizing bias in reviews.
  4.2 *Iterative* planning is more common in reviews that are exploratory in nature.
5. *Coverage.*
  5.1 *Exhaustive.* Reviewers attempt to locate and include all studies on a particular topic. Absent clear eligibility criteria, an exhaustive search may be overly broad and inefficient. Hence, many reviewers attempt to find all studies that meet *a priori* eligibility criteria. For example, Campbell and Cochrane intervention reviews pre‐specify characteristics of research participants, interventions, comparisons, outcomes, and study designs (PICOS) that will (and will not) be included in a review.
  5.2 *Representative.* Instead of locating all relevant research, reviewers obtain a representative sample of the studies conducted on a topic. This is difficult to do absent a sampling frame (exhaustive list) of all relevant studies.
  5.3 *Purposive.* Some reviewers choose to focus on central or pivotal works in an area, rather than an exhaustive or representative set of studies (Cooper, 1988). When this is done, reviewers should provide a clear rationale for the selection of studies.
  5.4 *Selective and incomplete*. **Rapid reviews** are selective and incomplete by design; thus, they should (and often do) include appropriate caveats about likely missing data.
  Reviews that purport to be exhaustive but only cite selected works are difficult for readers to evaluate and, in this sense, they are incomplete. Reviews that are limited to published studies are likely to be affected by reporting and publication biases, particularly if these reviews relate to intervention effects or other forms of hypothesis testing.
6. *Data types.* Reviews can include multiple sources of data, including:
  6.1 Quantitative primary studies (randomized controlled trials, cohort studies)
  6.2 Qualitative primary studies provide raw material for **qualitative evidence synthesis**.
  6.3 Individual participant data.
  6.4 Previous reviews. Reviews of reviews are often called **overviews.**
7. *Analysis and synthesis methods* used to configure and/or aggregate information:
  7.1 Narrative. Virtually all reviews include narrative summaries.
  7.2 Tabular. Tables are used to organize and display information (e.g., on characteristics of included studies, results of risk of bias assessments, summary of findings, other data matrices, evidence and gap maps).
  7.3 Graphic displays (e.g., PRISMA flow charts, forest plots, funnel plots, visual illustrations of theories of change).
  7.4 Qualitative methods include content analysis, critical interpretive synthesis, thematic synthesis, and qualitative comparative analysis (see Table 2).
  7.5 Pseudo‐statistical vote‐counting.
  7.6 Statistical **meta‐analysis** includes techniques for handling effect size multiplicity and pooling results across studies (generating point estimates, confidence intervals, and prediction intervals), assessing heterogeneity, moderator analysis (ANOVA analog, meta‐regression), **network meta‐analysis**, SROC curves, multivariate (SEM) models, hierarchical linear models (HLM), assessment of small sample and publication bias, and other sensitivity analyses.
  7.7 Economic analyses focus on service costs, cost‐effectiveness, or cost‐benefit comparisons.
8. Organization (Cooper, 1988)
  8.1 Historical
  8.2 Conceptual
  8.3 Methodological
9. Updating plans
  9.1 *Periodic.* Updates are often planned to occur every few years, but this may depend on the availability of resources and new data.
  9.2 *Ongoing.***Living systematic reviews** are continually updated, incorporating relevant new evidence as it becomes available (Elliott, Synnot, Turner, Simmonds, Akl, McDonald, et al., 2017).
10. Products designed for different audiences
  10.1 Plain language summary
  10.2 Brief report
  10.3 Full technical report

**Table 3:** Topics and questions for evidence synthesis

	Domains					
	**Conditions**		**Interventions**		**Methodology**	
**Topics**	Topics	Sample Questions	Topics	Sample Questions	Topics	Sample Questions
Constructs, categories	Assessment, diagnosis	How is Y defined, viewed, or assessed in different contexts?	Treatment types, processes, implementation	Which interventions are provided for Y problem? How are these interventions implemented?	Construct validity, diagnostic test accuracy	What are the properties of measurement instruments? (reliability, validity, sensitivity, specificity, underlying dimensions)
		What are different types of Y? experiences of Y?		How do interventions differ?		
Rates, trends	Incidence, prevalence	How often? How much? What is the incidence or prevalence of Y?	Treatment participation, acceptability	Attendance, retention, drop‐out, completion	Trend analyses	How to analysis and synthesis of data on rates and trends?
		Has incidence or prevalence of Y changed over time, place, context, subgroup?		Has attendance/retention changed over time?	Time series analyses	How to analyze and synthesize longitudinal, event history data?
			Treatment costs	What are the costs of X treatment?	Cost analyses	How to analyze and synthesize cost data?
Associations	Correlates	What is the relationship between X and Y?	Treatment correlates	What characteristics are associated with program completion?	Bivariate and multivariate models	How to analyze and synthesize data on associations?
Predictions	Risk and protective factors	To what extent does X predict Y?	Outcomes	Do initial client characteristics predict attendance or completion of treatment?	Prognostic test accuracy	What are the properties of a prognostic test? (predictive validity, sensitivity, specificity)
Causes and effects	Etiology	Does an increase in X cause an increase in Y?	Impact, efficacy, effectiveness	What are the effects of X treatment on Y outcomes?	Internal validity	How well do various methods control for threats to internal validity, rule out alternative plausible explanations, reduce risk of bias or error?
			Comparative effectiveness	What are the relative effects of X1 treatment vs X2 treatment on Y outcomes?	Design effects	How do elements of research design relate to results?
	Causal pathways	What direct and indirect pathways affect Y?	Moderators, mediators	Theories of change, moderator analysis	Complex models	How to synthesize data from complex networks or causal models?
			Cost effectiveness	Is one treatment more/less cost‐effective than another?	Economic analyses	How to estimate cost‐effectiveness, cost‐benefit?
Applications	Generaliz‐ability	How well do patterns hold up across samples, settings, or contexts?	Replication, transportability	Are intervention effects replicable? Is intervention transportable?	External validity	To what extent do measures or methods developed in one situation apply to others?

**Table 4:** Logic and evidentiary requirements for different kinds of inferences

	Logic and evidentiary requirements					
Inferences	Reliable and valid measures or thick description	Counts, ratios, proportions, or scales	Measures of association, statistical power	Time order	Theory of change, ability to rule out other plausible explanations	Probability sampling, inferential statistics, or proximal similarity
Constructs, categories	X					
Rates, trends	X	X				
Associations	X	X	X			
Predictions	X	X	X	X		
Causes and effects	X	X	X	X	X	
Generaliz‐ability	X	‐	‐	‐	‐	X

‐ depends on types of inferences that are generalized

Not included in this classification scheme are issues of philosophy of science and epistemology. Following Morgan (2007), who emphasized the importance of abduction (moving back and forth between inductive and deductive reasoning), inter‐subjectivity, and transferability, I believe that a pragmatic approach is needed to promote more robust and integrated synthesis methodologies. Toward that end, the classification scheme advanced here supports methodological pluralism, which is based on the premise that the “best” method for any particular study or review depends on the questions one is asking and the aims of the project. Far from an “anything goes” approach, methodological pluralism advances the value of goodness‐of‐fit between questions and methods. Thus, for example, the logic and evidence needed to support causal inferences differ from the logic and evidence needed to establish associations or generalizability (Table 4).

As noted above, I emboldened some terms that are commonly used to describe research reviews. The location of these terms within a broader framework illustrates the incomplete and idiosyncratic ways in which reviews are described: Some reviews are distinguished by their goals (scoping reviews and evidence and gap maps configure information, but do not (usually) aggregate results; meta‐analyses aggregate information; while systematic reviews are usually designed to maintain a neutral stance by minimizing bias and error). Other reviews are characterized by their coverage (rapid reviews), the type of information they use (qualitative evidence synthesis, individual participant data, and overviews), analytic methods (network meta‐analysis), or updating plans (living systematic reviews). A comprehensive framework is needed to fully describe the foci, goals, and methods of research reviews.

## CONCLUSIONS

By classifying research reviews, we aim to provide a conceptual scaffolding that can be used to describe available options, enhance communication about the types of reviews that are desired (e.g., when commissioning new reviews), support development of new reviews, and assess qualities of completed reviews. It is hoped that this can be useful for funders, review authors, consumers, and other stakeholders.

## Appendix

1. Domains
  1.1. Conditions
  1.2. Interventions
  1.3. Methodology
2. Topics
  2.1. Constructs and categories
  2.2. Rates and trends
  2.3. Associations
  2.4. Predictions
  2.5. Causes and effects
  2.6. Applications
3. Goals
  3.1. Configure information
  3.2. Aggregate or synthesize results
  3.3. Appraise study qualities
  3.4. Maintain a neutral stance or espouse a position
  3.5. Generate, explore, or test theory or hypotheses
  3.6. Inform practice, policy, and/or further research
4. Planning
  4.1. A priori
  4.2. Iterative
5. Coverage
  5.1. Exhaustive
  5.2. Representative
  5.3. Purposive
  5.4. Selective and incomplete
6. Data types
  6.1. Quantitative primary studies
  6.2. Qualitative primary studies
  6.3. Individual participant data
  6.4. Previous reviews
7. Analysis and synthesis methods
  7.1. Narrative
  7.2. Tabular
  7.3. Graphic
  7.4. Qualitative
  7.5. Pseudo‐statistical vote‐counting
  7.6. Statistical meta‐analysis
  7.7. Economic
8. Organization
  8.1. Historical
  8.2. Conceptual
  8.3. Methodological
9. Updating plans
  9.1. Periodic
  9.2. Ongoing
10. Products
  10.1. Plain language summary
  10.2. Brief report
  10.3. Full technical report
